# A medoid-based deviation ratio index to determine the number of clusters in a dataset

**DOI:** 10.1016/j.mex.2023.102084

**Published:** 2023-02-25

**Authors:** Kariyam, Abdurakhman, Adhitya Ronnie Effendie

**Affiliations:** a Department of Mathematics, Faculty of Mathematics and Natural Sciences, Gadjah Mada University, Indonesia; b Department of Statistics, Faculty of Mathematics and Natural Sciences, Universitas Islam Indonesia, Indonesia

**Keywords:** The number of clusters, Deviation ratio index, K-medoids based on block deviation, *Medoid-based deviation ratio index to determine the number of clusters*

## Abstract

Most existing methods of determining the number of groups apply to particular data types or are calculated based on the distance matrix for all object pairs. In this paper, we propose a medoid-based Deviation Ratio Index (DRI) to determine the number of clusters. The DRI is calculated based on the distance matrix for each object to k final medoids. These final medoids are produced by the block-based k-medoids algorithm (BlockD-KM). We choose a specific transformation and a suitable distance for certain variables before executing the BlockD-KM. We illustrated the detailed stages of DRI on secondary data in the 2022 environmental index of Asia Pacific countries, so that they are easy to reproduce. We use eight real datasets, namely Breast Cancer, Heart Disease, Iris, Wine, Soybean, Ionosphere, Vote, and Credit Approval data, to validate the DRI method. We compare the DRI method with the Calinski-Harabaz (CH) and the Silhouette index. The experimental results show that the DRI is 100% correct in predicting the number of clusters. While the CH index correctly predicts 62.5% and the Silhouette index of 75%. We also generated three kinds of artificial data to evaluate the proposed method, and 76.7% of the experiments were predicted correctly.

• The medoid-based deviation ratio index aids the researcher in determining the number of clusters
• The DRI method applicable to any medoids-based partitioning algorithm
• This method is suitable for all data types (categorical, numerical, and mixed)

## Graphical abstract

**Figure:**
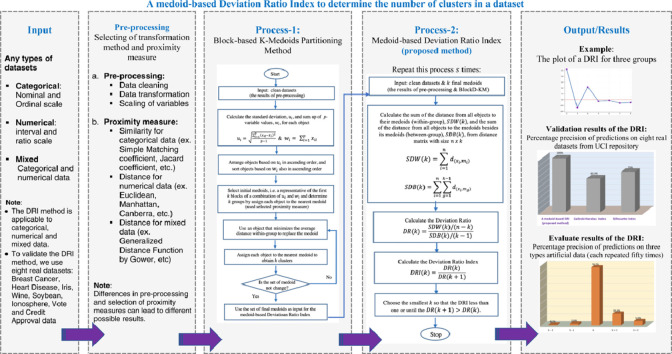

Specifications Table

**Table:** 

Subject area	Mathematics and Statistics
More specific subject area	*Medoid-based Clustering*
Name of your method	*Medoid-based deviation ratio index to determine the number of clusters*
Name and reference of original method:	• T. Calinski and J. Harabaz, A dendrite method for cluster analysis, Communications in Statistics, 3(1) (1974) 1–27. http://dx.doi.org/10.1080/03610927408827101 • P.J. Rousseeuw, Silhouettes: a graphical aid to the interpretation and validation of cluster analysis, Journal of Computational and Applied Mathematics, 20 (1987) 53–65. DOI: 10.1016/0377-0427(87)90125-7 • H.S. Park and C.H. Jun, A Simple and Fast Algorithm for K-Medoids Clustering. Expert System with Applications, 36(2) (2009), 3336–3341. https://doi.org/10.1016/j.eswa.2008.01.039
	• Kariyam, Abdurakhman, Subanar, Utami, H., Effendie, A.R. Block-based K-medoids partitioning method with standardized data to improve clustering accuracy, Mathematical Modelling of Engineering Problems, 9(6) (2022), 1613–1621. https://doi.org/10.18280/mmep.090622
Resource availability:	https://epi.yale.edu/downloads/epi2022report06062022.pdf • Chapter 5 Table 5–2 page 71: Regional rankings and score on Air Quality • Chapter 6 Table 6–2 page 80: Regional rankings and score on Sanitation and Drinking Water • Chapter 7 Table 7–2 page 87: Regional rankings and score on Heavy Metals • Chapter 8 Table 8–2 page 95: Regional rankings and score on Waste Management

## Method details

### Background

Cluster analysis is a statistical method used to group similar objects based on their characteristics or features. The high homogeneity within groups and high heterogeneity between groups indicate the good result of a cluster analysis. Several techniques are used in cluster analysis: centroid-based, hierarchical, density-based, distribution-based, subspace and projection-based, and constraint-base. Each method has its strengths and weakness and is suited to different data types and applications.

Estimating the number of clusters is an essential step in cluster analysis as it helps determine the appropriate granularity level for the grouping. Choosing a large group size will make the group members small. In contrast, if we choose a small group size, it will make the large group members. Accurately estimating the number of groups is essential for the success of the analysis, as it impacts the validity and reliability of results. It can also help identify patterns and relationships within the data and be used to make informed decisions and predictions about it. There are several methods for estimating the number of clusters, such as Latent Class Cluster Analysis (LCCA) [1], Silhouette index [2], Silhouette with Principle Component Analysis (PCA) [3], Fuzzy C-means (FCM) [4], the Cubic Clustering Criterion (CCC) [5], algorithm based on dichotomy [6], gap statistics [7], Krzanowski-Lai (KL) index [8], Calinski-Harabaz (CH) index [9], and so on.

The LCCA estimates latent cluster membership based on model fit, cluster separation, and partition stability. This method is limited to categorical variables with assumed multinomial distributions. The Silhouette index evaluates the similarity of each object to its cluster compared to other groups. This index calculates a Silhouette score for each point. A value close to 1 indicates that the object is well-matched to its cluster. While a value close to −1 indicates that it may have been assigned to the wrong group [2]. Silhouette index can be used for any number of clusters, but it can be sensitive to the choice of the distance matrix. Then Silhouette based on PCA and an algorithm based on dichotomy use of the k-means algorithm. Even though the k-means algorithm is a popular algorithm for partitioning a dataset, this method is sensitive to initial conditions and unsuitable for non-numeric data or categorical variables. Meanwhile, the FCM algorithm uses fuzzy logic to automatically determine the number of clusters in a dataset. The FCM works by minimizing the objective function, which is defined as the sum of the squared distances between data points and their nearest cluster centers. The FCM algorithm can be sensitive to the initial values of the cluster centers and may be computationally expensive, especially for large datasets with many features. The Cubic Clustering Criterion uses the Monte Carlo study as a "stopping rule" to determine the number of clusters. Then the Gap Statistic compares the Within-Clusters Sum of Square (WCSS) of the observed data to that of a reference dataset generated by sampling. The optimal number of groups is the point at which the gap between the WCSS of the observed data and the reference dataset is the largest [6]. The Gap Statistic is computationally intensive and may not work well for small datasets. Similar to gap statistics the Krzanowski-Lai index also consider the sampling results of data. The KL index compares the observed data to a reference dataset generated by sampling the data and calculates a KL index for each number of clusters. The optimal number of clusters is the point at which the KL index is the highest. The KL index is also unsuitable for all data types; for example, it is not recommended for binary data. Furthermore, the Calinski-Harabaz index, also known as the Variance Ratio Criterion (VRC), is a method that can be used in conjunction with a dendrogram to determine the number of clusters. The CH index is calculated based on the ratio of the between-cluster variance to the within-cluster variance. The higher the CH index, the better the clustering solution. This index is a widely used and well-established method for evaluating the quality of clustering solutions and is relatively simple to calculate. However, the CH index is sensitive to the scale of the variables meaning that the results may be affected by the units of measurement. If the CH index is executed based on the proximity matrix of the size of nxn, then it may not work well for big data. It can be concluded that these methods are suitable for specific conditions, whether related to data types or data sizes or certain grouping methods. Therefore we will develop a method that can overcome several of these problems simultaneously.

This research aims to develop a more flexible alternative method to determine the number of clusters. We introduce a new method, namely a medoid-based Deviation Ratio Index (DRI), to determine the number of clusters in a dataset. The newly proposed method is executed based on the proximity matrix of size nxk, i.e. the distance of all objects to k final medoids. We choose the k-medoids algorithm because it is robust to noise and outliers. K-medoids clustering, part of the centroid-based technique, is an algorithm for partitioning a set of n objects into k clusters, where each cluster is represented by one of the n objects, called a medoid. The medoid of a group is the most central object with the smallest average distance from other entities. The k-medoids algorithm minimizes the sum of dissimilarities between each data point and its closest medoid. This algorithm can also handle non-numeric data and dissimilarity measures so that it can be used in various fields. The medoid-based DRI provides another perspective to determine the number of clusters of any kind, whether categorical, numerical or mixed data. We hope the newly proposed method significantly contributes to the literature on the clustering technique.

### Data types in cluster analysis and datasets to validate the medoid-based Deviation Ratio Index

There are four scale data types in cluster analysis: nominal, ordinal, interval, and ratio scale [10]. Cluster analysis can also be applied to text, images, graphs, and time series. The proposed method is limited to data with categorical (nominal and ordinal), numerical (interval and ratio), and mixed data. The mixed data consists of nominal and ordinal, nominal and numerical, ordinal and numerical, and a mixture of the three, namely nominal, ordinal and numeric.

In this paper, we implement three kinds of data to explain and evaluate the proposed method. To explain in detail how to run our proposed method, we use secondary data about the environmental health of Asia Pacific. These data consist of 25 countries observed on four environmental issues with numerical data, namely Air Quality (AQ), Water and Sanitation (WS), Heavy Metals (HM), and Waste Management (WM) scores [11]. Then we apply eight real datasets to evaluate the proposed method, i.e. Breast Cancer data, Heart Disease data, Iris data, Wine data, Soybean (small) data, Ionosphere data, Vote data, and Credit Approval data [12]. The Breast Cancer and Heart Disease datasets are well-known in medical research and pattern recognition. The Breast Cancer dataset contains 569 samples of Breast Cancer tissue, each with 30 numerical variables related to the characteristics of the tissue. The dataset is commonly used for classification tasks and contains two classes of breast cancer tissue: malignant and benign. Meanwhile, the Heart Disease dataset consists of 303 samples of patients, each with five numerical and eight categorical features related to their medical history and examination results. The dataset contains two classes of patients: those with heart disease and those without heart disease. The Iris and Wine datasets are well-known in pattern recognition and multivariate statistics. It contains 150 samples of Iris flowers, each with four features: sepal length, sepal width, petal length, and petal width. The dataset is commonly used for classification tasks, and it contains three clusters of iris flowers: Iris Setosa, Iris Virginica, and Iris Versicolor. The Wine dataset contains 178 samples, each with 13 numerical features related to the chemical properties of the wine. This data is grouped into three classes. The Soybean (small) dataset is well-known in agriculture and pattern recognition. It contains 47 samples of soybean plants, each with 35 binary features indicating the presence or absence of certain disease symptoms and grouping into four classes. The Ionosphere dataset is usually used in the field of pattern recognition and signal processing. This data consists of 351 samples of radar return signals, each with 34 numerical variables related to the properties of the signal. The dataset contains two clusters of radar signals: good and bad. Furthermore, the Vote dataset is data about political science and pattern recognition. These data involve 232 (non-missing) house of representative members of congress with 16 key votes binary attributes. The last dataset, Credit Approval data, is a well-known dataset in the field of finance and pattern recognition. It contains 653 non-missing samples of credit applications, each with nine binary and six continuous variables related to the applicant's credit history and financial situation. The dataset contains two groups of credit applications: those that were approved and those that were denied. All real datasets are publicly available in the University of California, Irvine (UCI) Machine Learning Repository. The summary of eight real datasets is in Table 1.

**Table 1:** The profile of the eight real datasets to validate the medoid-based Deviation Ratio Index.

No	Datasets	Type	n	The true number of groups, k	Variables	
					$p_{c}$	$p_{n}$
(1)	(2)	(3)	(4)	(5)	(6)	(7)
1	Breast cancer	Numerical	569	2	–	30
2	Wine	Numerical	178	3	–	13
3	Iris	Numerical	150	3	–	4
4	Ionosphere	Numerical	351	2	–	33
5	Soybean	Categorical	47	4	35	–
6	Vote	Categorical	232	2	16	–
7	Heart disease	Mixed	303	2	8	5
8	Credit approval	Mixed	653	2	9	6

n: number of objects; k: number of actual clusters; $p_{c}$: number of categorical variables; $p_{n}$: number of numerical variables.

Additionally, we created three various artificial datasets and executed each of them fifty times. This experiment is intended to evaluate the performance of the medoid-based Deviation Ratio Index (proposed method).

### Pre-processing for the medoid-based Deviation Ratio Index

Pre-processing data is an essential stage in cluster analysis as it can significantly affect the results. There are different ways of pre-processing data; the best way will depend on the specific data and clustering algorithm used. Pre-processing typically includes cleaning, transforming, and scaling the data to ensure that it is in a suitable format for the clustering algorithm to work effectively. Data cleaning involves removing missing or incorrect values and dealing with outliers. Meanwhile, data transformation can include normalizing or standardizing the data or converting categorical variables into numerical ones. Scaling is also an important step in pre-processing, which ensures that all the dataset variables are on the same scale. This process makes it possible to compare the variables and make the data more suitable for clustering algorithms. We suggested pre-processing in a standardized form to achieve comparability in numerical or mixed data. We will obtain the unification from this preprocessing. Eighteen methods for standardizing variables in cluster analysis have been investigated based on distance and normalized data matrix [13].

The general normalization of numerical data are linear transformations with a standardization formula as follows [14],

(1) $$z_{ij}=bx_{ij}+a\;\left(b>0\right)$$

where $x_{ij}\left(z_{ij}\right)$ denotes the value (standardized value) of the j-th variable for i-th object. The value that can be used in cluster analysis is $b=\frac{1}{max\;\left(x_{j}\right)-min\;\left(x_{j}\right)}$ and $a=-\frac{min\;(x_{j})}{max\;\left(x_{j}\right)-min\;\left(x_{j}\right)}$, so that Eq. (1) can be rewritten as follows,

(2) $$Z_{ij}=\frac{x_{ij}\;-\;min\left(x_{j}\right)\;}{max\;\left(x_{j}\right)-min\;\left(x_{j}\right)}$$

For ordinal data, Reference [10] page 30 suggests the formula such as below,

(3) $$Z_{ij}=\frac{r_{ij}\;-\;1\;}{M_{j}-1}$$

where $r_{ij}$ is a rank of the *i* th object in a *j-*th variable and $M_{j}$ is the highest rank of a *j-*th variable.

### Proximity measure for the medoid-based Deviation Ratio Index

Proximity measure is a method used in cluster analysis to quantify the similarity or distance between objects in a dataset. It is important to note that some of the proximity measures are sensitive to the scale of the variables, and it is necessary to standardize the data before applying the distance or similarity measure. The choice of proximity measure will depend on the specific data and the type of clustering algorithm being used. There are some similarities between objects for binary data, such as the Simple Matching coefficient, Jacard coefficient, Rogers and Tanimoto coefficient, Sneath and Sokal coefficient, Gower and Legendre coefficient, and others. Euclidean distance, Manhattan distance, Minkowski distance, Pearson correlation, and Canberra distance are commonly used as proximity measures for continuous variables [10,15].

The similarity measure for categorical data with more than two levels could be dealt with similarly to binary data, with each level of a variable being regarded as a single binary variable [15]. A method is to allocate a score $s_{ijk}$ of zero or one to each variable k, depending on whether the two objects i and j are the same on that variable. These scores are then simply averaged over all p variables to give the required similarity coefficient, such as Reference [15] page 48 below,

(4) $$s_{ij}=\frac{1}{p}\;\sum_{k=1}^{p}s_{ijk}$$

The Euclidean distance is formulated as follows [15],

(5) $$d_{ij}={\left[\sum_{l=1}^{p}{\left(x_{il}-x_{jl}\right)}^{2}\right]}^{\frac{1}{2}}\;i=1,2,\;\ldots ,n,\;j=1,2,...,n$$

where $d_{ij}$ is distance object i and object j. While the Canberra distance is formulated as follows [15],

(6) $$d_{ij}=\sum_{l=1}^{p}\frac{\left|x_{il}-x_{jl}\right|}{\left|x_{il}\right|+\left|x_{jl}\right|}$$

Furthermore, a generalized distance function (GDF) between object i and j for non-missing mixed data as follows [10,15],

(7) $$d_{ij}={\left(\alpha \sum_{s=1}^{p_{b}}\delta_{b}\left(x_{is},x_{js}\right)+\beta \sum_{t=1}^{p_{c}}\delta_{c}\left(x_{it},x_{jt}\right)+\gamma \sum_{q=1}^{p_{n}}\delta_{n}\left(x_{iq},x_{jq}\right)\right)}^{\omega }$$

where α, β, and γ are the weights for the binary, categorical, and numerical variables, and ω is the weight for the whole function, respectively. In this paper, we apply the same weight, i.e. α=β=γ=ω=1.

### Medoids-based partitioning method for the medoid-based Deviation Ratio Index

The new method, a medoid-based Deviation Ratio Index to determine the number of clusters, is flexible for any medoid-based partitioning method. Since Kaufman and Rousseuuw first introduced the Partitioning Around Medoids (PAM) or often called the K-Medoids (KM) algorithm, in 1987 [10], this method has inspired many researchers to improve the performance of KM. Some of them are simple and fast KM [16], ranked KM [17], simple KM [18], initialization of the flexible KM using deviation [19], fast and eager KM [20], minimization of the number of iterations in KM [21], crow search algorithm of KM [22], block-based KM with standardized data [23] and many more. In this paper, we use k-medoids based on the block of the deviation and the sum of p variable values (BlockD-KM). In the BlockD-KM, we substitute the first phase of simple and fast KM [16] with the first stage of the flexible KM [19]. The procedure of the BlockD-KM is as follows [23].

1. Calculate the sum up of p-variables values and standard deviation of p-variables for each object, i, (i=1,2,⋯,n),such as below,
  (8) $$u_{i}=\sqrt{\frac{\sum_{l=1}^{p}{\left(x_{il}-{\bar{x}}_{i}\right)}^{2}}{p-1}}$$
  where ${\bar{x}}_{i}=w_{i}/p;$ with $w_{i}$ is sum up of p-variables values as follows,
  (9) $$w_{i}=\;\sum_{l=1}^{p}x_{il}$$
  where i=1,2,⋯,n; l=1,2,⋯,p.
2. Sort objects in ascending order, first based on Eq. (8), $u_{i},$ then each block of the identical standard deviation (if any), arrange objects based on Eq. (9), $w_{i}$, also in ascending order.
3. Select the first object as initial medoids from the first k blocks of the combination of $u_{i}$ and $w_{i}$ (or may only block of $u_{i}$).
4. Determine the members of kinitial groups based on the distance of an object to the nearest medoid.
5. Update the current medoid in each cluster based on the object that minimizes the average distance to other things in its group. The average distance within cluster *g-*th, which has $n_{g}$ members for an object *i* th, ${\bar{D}}_{i},$ defined as follows,
  (10) $${\bar{D}}_{i}=\frac{1}{n_{g}}\;\sum_{j=1}^{n_{g}}d_{ij}$$
6. Obtain the cluster by assigning each object to the closest medoid. Then calculate the total deviation within groups or the sum of the distance from all things to their medoids, SDW(k), such as below,
  (11) $$SDW\left(k\right)=\sum_{i=1}^{n}d_{\left(x_{i},m_{i}\right)}$$

with $m_{i}$ is a medoid of the group containing an object $x_{i}.$

7. Repeat steps 5 and 6 until the SDW(k) is equal to the previous one or the set of medoids does not change, or a pre-determined number of iterations is reached.

Even though the medoid-based deviation ratio index is designed for the k-medoids algorithm, we can apply it to other clustering methods by first finding the central object as a medoid. To obtain the medoid, we can run it easily, based on things with the smallest average distance in the group.

### Validation in cluster analysis and validation for the medoid-based Deviation Ratio Index

One method for validation in cluster analysis is external validation. External validation is a method used to evaluate the quality and performance of clustering results. External validation measures the similarity between the clusters obtained by the clustering algorithm and the true class labels of the data if they are available [24]. This value can be done by comparing the clusters obtained by the algorithm to the true cluster labels using measures such as clustering accuracy, Fowlkes-Mallows index or adjusted Rand index. The value range is between 0 and 1, with 1 indicating a perfect match between the clusters and the true class labels. The larger this value, the better the accuracy. The clustering accuracy is defined as follows [25]

(12) $$Acc=\frac{1}{n}\sum_{g=1}^{k}a_{g}$$

where nis the number of objects; k is the number of clusters; and $a_{g}$ is the number of objects in considered groups correctly assigned to the actual clusters.

Furthermore, we also compare the result of our proposed method with others. We use the Variance Ratio Criterion based on distance [9] and the Silhouette index [2] as comparisons. The widely used formula VRC (often called CH index) to determine the number of clusters such as below [7],

(13) $$CH\left(k\right)=\frac{trace\left(B_{k}\right)/\left(k-1\right)}{trace\left(W_{k}\right)\;\left(n-k\right)},$$

where $B_{k}$ and $W_{k}$ are matrix of between-cluster and within-cluster sum of squares errors. The Calinski-Harabaz also formulated the VRC based on the distance matrix as below [9].

(14) $$VRC=\frac{BGSS/\left(k-1\right)}{WGSS/\left(n-k\right)}=\frac{\left({\bar{d}}^{2}+A_{k}\left(n-k\right)/\left(k-1\right)\right)}{\left({\bar{d}}^{2}-A_{k}\right)}$$

where

(15) $$WGSS=\frac{1}{2}\left(\left(n_{1}-1\right){\bar{d}}_{1}^{2}+\left(n_{2}-1\right){\bar{d}}_{2}^{2}+\cdots +\left(n_{k}-1\right){\bar{d}}_{k}^{2}\right)$$

and

(16) $$BGSS=\frac{1}{2}\left(\left(k-1\right){\bar{d}}^{2}+\left(n-k\right)A_{k}\right)$$

where

(17) $$A_{k}=\frac{1}{n-k}\;\left(\left(n_{1}-1\right)\left({\bar{d}}^{2}-{\bar{d}}_{1}^{2}\right)+\left(n_{2}-1\right)\left({\bar{d}}^{2}-{\bar{d}}_{2}^{2}\right)+\cdots +\left(n_{k}-1\right)\left({\bar{d}}^{2}-{\bar{d}}_{k}^{2}\right)\right)$$

is a weighted mean of the difference between the general and the within-group mean squared distances.

For observation i, let a(i) be the average distance of object i to all entities within the cluster, and b(i) the average distance of object i to points in the nearest groups besides its own closest is defined by the group minimizing this average distance. Then the silhouette index can be calculated via [2]

(18) $$s\left(i\right)=\frac{b\left(i\right)-a\left(i\right)}{max\left\{a\left(i\right).b\left(i\right)\right\}}$$

A point is well clustered if s(i) is large, and the best-separated clusters have an index equal to one. The group size that produces the highest average silhouette index is chosen as the best number of clusters. Meanwhile, the medoid-based shadow value (MSV) for an object x is defined such as below [26],

(19) $$msv\left(x\right)=\frac{d\left(x,\;m^{\prime }\left(x\right)\right)-d\left(x,m\left(x\right)\right)}{d(x,\;m^{\prime }\left(x\right)}$$

where d(x,m(x)) is the distance between object x to the first closest medoid and $d(x,m^{\prime }\left(x\right))$ is the distance between object x to the second closest medoid.

### A medoid-based Deviation Ratio Index to determine the number of clusters (proposed method)

We introduce a novelty procedure to determine the number of clusters in a dataset through the medoid-based deviation ratio index. We were inspired by the Variance Ratio Criterion (VRC), which uses an object distance matrix of all pairs of objects as a basis to derive the VRC [7]. We also consider the medoid-based shadow value (MSV) concept that implements the first and second closest centroid to develop the MSV [26]. Meanwhile, the MSV adapt the Silhouette index [2] and centroid-based shadow value. We only use a distance matrix with size nxk, i.e. the distance of all objects to each final medoid, to construct our proposed method. Suppose there are n objects with observations on the same p variables for each individual and separated into k groups. Based on final medoids for a specific size of clusters, k, the deviation ratio, DR(k), is formulated as follows.

(20) $$DR\left(k\right)=\frac{SDW\left(k\right)/\left(n-k\right)}{SDB\left(k\right)/\left(k-1\right)}$$

where SDW(k) is the sum of the distance from all objects to their medoids (within-group), such as Eq. (11). While SDB(k) is the sum of the distance from all objects to the medoids besides its medoids (between-group). The formula of SDB(k) is as follows,

(21) $$SDB\left(k\right)=\sum_{i=1}^{n}\sum_{g=1}^{k-1}d_{\left(x_{i},m_{g}\right)}$$

where $m_{g}$ is the medoids of the other group.

Furthermore, the deviation ratio index (DRI(k)) is defined as the comparison of the deviation ratio of a cluster of size k to a group of size (k+1). The deviation ratio index for a group with a size of k is formulated as follows,

(22) $$DRI\left(k\right)=\frac{DR\left(k\right)}{DR\left(k+1\right)}$$

The optimal number of groups is determined as the smallest k so that the deviation ratio index, DRI(k), is less than one. Another way we may start with k=2 and add a cluster until the value of the DR(k) is less than DR(k+1) or DR(k)<DR(k+1). This way can be faster than the fuzzy C-means (FCM) clustering algorithm. The FCM algorithm uses the range between two and $int(\sqrt{n})$ as a basis for selecting the optimal number of groups [4]. The reason we formulate DR(k) using SDW(k) as the quantifier is to anticipate extreme data. The intended data extreme is that each group is perfectly separated, so the value of SDW(k) is zero. Meanwhile, if the group size increases, there is a tendency for deviation within groups, SDW(k), to decrease. At the same time, the value of SDB(k) will tend to get bigger. According to this fact, we formulate the DRI(k) as a comparison between the DR(k) of group k and the DR(k+1) of one larger group. Therefore, based on the parsimony principle, the best group size is the first smallest group size with a DRI(k) value of less than one. This index is not defined for k=1.

### An illustrative example

The primary purpose of presenting the following examples is to clarify the implementation of our proposed method, making it easy to replicate on any data set. We use secondary data from Reference [11] on the sub-section of policy about environmental health in Asia Pacific regions. We will group it into three clusters. In this example we subtract the data with the smallest data and then divide it by its range to standardize data as Eq. (2) [14]. The results of standardizing for four environmental health scores, i.e. air quality (AQ), water and sanitation (WS), heavy metals (HM), and waste management (WM) scores [11], are in columns (3) to (6) in Table 2. Then we apply the k-medoids based on block deviation (BlockD-KM).

**Table 2:** An example of the summary process to calculate the DR(k) for k=3.

No	Country code	Standardized data for four issues (variables) in the environmental health				K-medoids based on block-deviation					The criterion of deviation ratio index				
						The process to get initial medoids			Iteration to get final medoids		Distance objects to the final medoids			The criterion for DR(k)	
		AQ	WS	HM	WM	Std dev	Sum	IG	I1	I2=I3	$d_{\left(x_{i},o_{1}\right)}$	$d_{\left(x_{i},o_{13}\right)}$	$d_{\left(x_{i},o_{3}\right)}$	min$\;d_{i}$	$\;b_{i}$
(1)	(2)	(3)	(4)	(5)	(6)	(7)	(8)	(9)	(10)	(11)	(12)	(13)	(14)	(15)	(16)
1	TLS	0.12	0.15	0.12	0.13	0.0158	0.53*	G1*	G1*	G1*	0.00	1.52	0.26	0.00	1.78
2	JPN	0.45	0.54	0.49	0.40	0.0573	1.87*	G2*	G3	G3	0.68	0.86	0.44	0.44	1.54
3	PHL	0.17	0.31	0.30	0.24	0.0628	1.02*	G3*	G3*	G3*	0.26	1.27	0.00	0.00	1.53
4	SOL	0.28	0.15	0.12	0.16	0.0693	0.70	G1	G1	G1	0.16	1.45	0.28	0.16	1.73
5	SKR	0.17	0.25	0.13	0.09	0.0699	0.63	G1	G3	G1	0.12	1.47	0.24	0.12	1.71
6	PNG	0.26	0.26	0.21	0.11	0.0743	0.84	G3	G3	G3	0.20	1.38	0.19	0.19	1.58
7	KHM	0.85	0.98	0.79	1.00	0.0985	3.61	G2	G2	G2	1.55	0.12	1.31	0.12	2.86
8	TWN	0.27	0.03	0.15	0.16	0.0987	0.61	G1	G1	G1	0.20	1.51	0.34	0.20	1.85
9	VNM	0.03	0.21	0.17	0.00	0.1014	0.41	G1	G1	G1	0.18	1.59	0.32	0.18	1.91
10	VUT	0.21	0.00	0.00	0.05	0.1017	0.26	G1	G1	G1	0.23	1.68	0.47	0.23	2.15
11	THA	0.10	0.18	0.12	0.33	0.1051	0.73	G1	G1	G1	0.20	1.41	0.25	0.20	1.66
12	MIC	0.31	0.22	0.28	0.48	0.1098	1.30	G3	G3	G3	0.43	1.14	0.29	0.29	1.57
13	CHN	0.75	0.95	0.85	1.00	0.1108	3.54	G2	G2	G2*	1.52	0.00	1.27	0.00	2.79
14	MNG	0.18	0.48	0.29	0.27	0.1242	1.23	G3	G3	G3	0.40	1.18	0.17	0.17	1.57
15	IDN	0.49	0.72	0.64	0.80	0.1326	2.65	G2	G2*	G2	1.08	0.45	0.84	0.45	1.92
16	FIJ	0.73	0.88	0.57	0.81	0.1350	2.99	G2	G2	G2	1.25	0.34	1.02	0.34	2.27
17	WSM	0.25	0.09	0.27	0.44	0.1441	1.05	G3	G3	G3	0.37	1.28	0.31	0.31	1.65
18	BRN	1.00	1.00	1.00	0.70	0.1505	3.70	G2	G2	G2	1.61	0.42	1.37	0.42	2.98
19	LAO	0.33	0.43	0.57	0.67	0.1511	2.01	G2	G2	G3	0.79	0.79	0.55	0.55	1.58
20	TON	0.00	0.36	0.10	0.12	0.1523	0.58	G1	G1	G1	0.24	1.50	0.29	0.24	1.79
21	KIR	0.42	0.40	0.71	0.37	0.1559	1.90	G2	G3	G3	0.74	0.91	0.50	0.50	1.65
22	SGP	0.26	0.02	0.46	0.28	0.1816	1.02	G3	G3	G3	0.42	1.33	0.35	0.35	1.75
23	MHL	0.24	0.25	0.68	0.40	0.2047	1.58	G2	G3	G3	0.64	1.06	0.43	0.43	1.70
24	MYS	0.30	0.52	0.74	0.32	0.2056	1.88	G2	G3	G3	0.76	0.93	0.51	0.51	1.69
25	MMR	0.09	0.56	0.16	0.32	0.2090	1.13	G3	G3	G3	0.45	1.23	0.31	0.31	1.68
						Total deviation		8.0	7.1	6.7	Sum deviation of (a) within & (b) between			6.7 (a)	46.9 (b)
											Deviation ratio DR(k=3)			0.013	

*medoids of groups.

Table 2 shows the summary of group enumerations based on the criterion of deviation ratio index. Steps 1 and 2 of BlockD-KM generate ordered objects based on the standard deviation, such as columns (2), (7) and (8). We intentionally display the standard deviation in four decimal places, such as column (7). This presentation is to make it easier to observe the difference of the first k smallest standard deviation of objects. We can ignore sorting the thing based on the sum of the data as the results in column (8). The reason is that none of the three entities with the first smallest standard deviation is identical. Then the step 3, based on the block of deviation, we obtain object one or Timor Leste (TLS), object two or Japan (JPN), and object three or Philippines (PHL) as initial medoids such as in column (8). According to the Euclidean distance of the object to the nearest medoid, steps 4 of BlockD-KM produces the initial groups (IG), such as column (9), with a total deviation or SDW(k) of 8.0.

To get the set of medoids that does not change, we update the current medoid of each group based on the object that minimizes the average distance to other things in its group. The first iteration in the fifth and sixth steps of the BlockD-KM does not change the medoid of group one and group three. Meanwhile, for group two, the object with the smallest average distance from members in the group is the 15th object or Indonesia (IDN). This new medoid causes a change in the members of each group as in column (10) with SDW(k) of 7.1. Then we update the medoid again, resulting from iteration one. In iteration two, the medoid for group two again changed from Indonesia (object 15th) to China (object 13th). While the medoids of groups one and three remained the same. The set of new medoids from iteration two produces the members of each group, such as column (11) withSDW(k) value of 6.7.

**Fig. 1:**
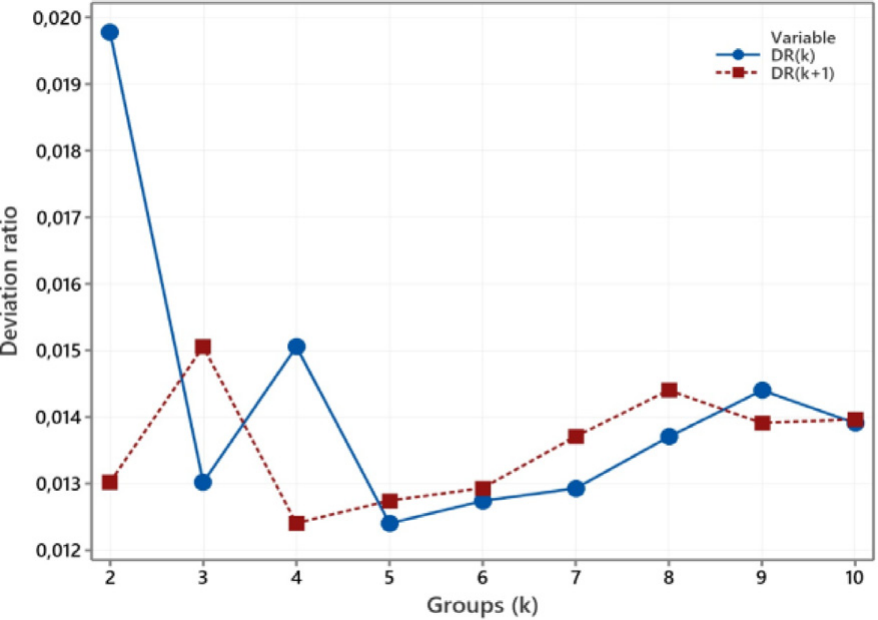
The plot of DR(k) and DR(k+1).

**Fig. 2:**
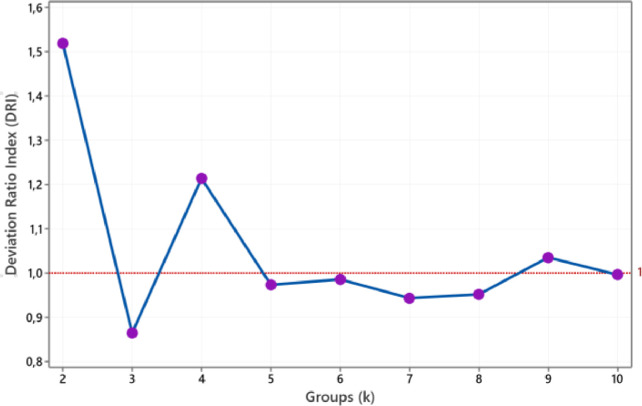
The plot of DRI(k).

In the same way, updating the medoid is continued in the third iteration by looking for objects with the smallest average distance to group members. Iteration three doesn't change the medoid, so the BlockD-KM stage is complete. This dataset needed three iterations to achieve an unchanged medoids as the final medoid. Furthermore, to obtain the criterion values of the deviation ratio, we calculate the SDW(k) value such as in column (15) and the SDB(k) value such as in column (16). According to Eq. (20), with the input SDW(k) value of 6.71 and SDB(k) value of 14.6, the deviation ratio is 0.013. In the same way, the values ofDR(k) and DR(k+1) for two to ten groups are shown in Fig. 1. Meanwhile, the deviation ratio index DRI(k) values are shown in Fig. 2. According to Fig. 1 or Fig. 2, the optimal number of clusters for environmental health data in Asia Pacific countries is three groups.

The members of the set final medoids are the first object $\left(O_{1}\right)$ Timor Leste (TLS), object three $\left(O_{3}\right)$ Philippines (PHL), and object thirteen $\left(O_{13}\right)$ China (CHN). Then based on the distance object to the closest final medoid, we obtain the members of each cluster. The first cluster consists of Timor Leste (TLS), Solomon Islands (SOL), South Korea (SKR), Taiwan (TWN), Viet Nam (VNM), Vanuatu (VUT), Thailand (THA), and Tonga (TON). The members of the second group are the states Cambodia (KHM), China (CHN), Indonesia (IDN), Fiji (FIJ), and Brunei Darussalam (BRN). The third group includes Japan (JPN), Philippines (PHL), Papua New Guinea (PNG), Micronesia (MIC), Mongolia (MNG), Samoa (WSM), Laos (LAO), Kiribati (KIR), Singapore (SGP), Marshall Islands (MHL), Malaysia (MYS), and Myanmar (MMR).

## Method validation

**Fig. 3:**
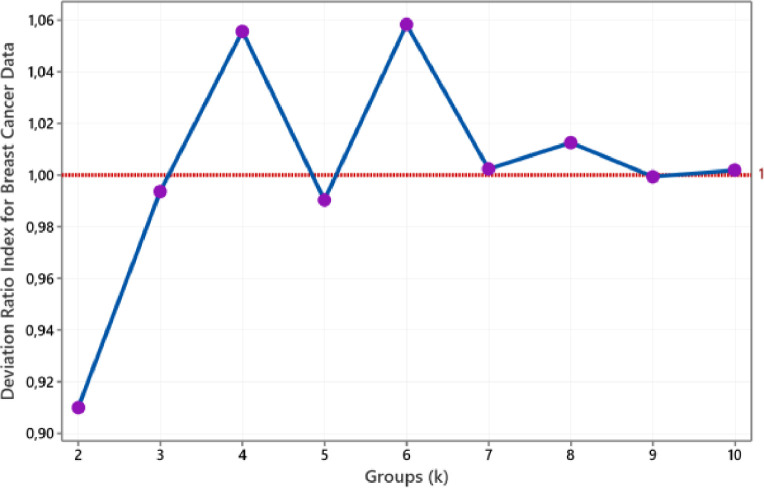
The plot of DRI for Breast Cancer data.

We use eight actual datasets to evaluate the proposed method. All real data sets taken from the machine learning repository of the University of California, Irvine (UCI), i.e. Breast Cancer data, Heart Disease data, Iris data, Wine data, Soybean (small) data, Ionosphere data, Vote data, and Credit Approval data [12]. As in an illustrative example, we use k-medoids based on block-deviation (BlockD-KM) to clusters of all datasets. Even though we can stop the process until the DR(k+1) is more than DR(k), in this paper, we execute by repeat procedure for two to ten clusters for each dataset. We applied the Euclidean distance for Breast Cancer and Wine data. For Iris and Ionosphere data, we implement Canberra distance. Then we use the Simple Matching distance for Vote and Soybean data. Meanwhile, we use the Gower distance for the Heart Disease and Credit Approval data. According to these constraints, we get the deviation ratio index for each dataset, such as Fig. 3 to Fig. 10.

**Fig. 4:**
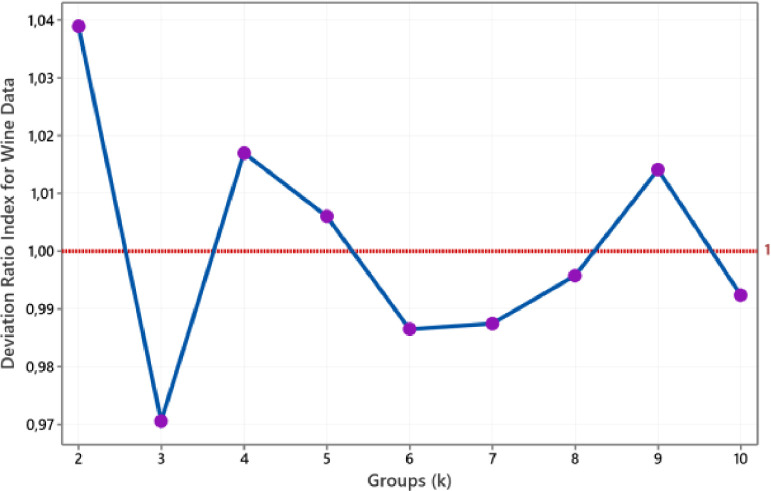
The plot of DRI for Wine data.

**Fig. 5:**
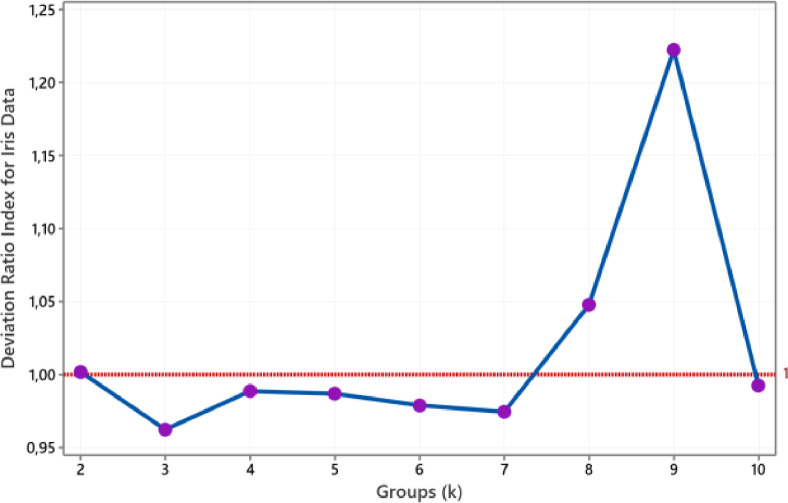
The plot of DRI for Iris data.

**Fig. 6:**
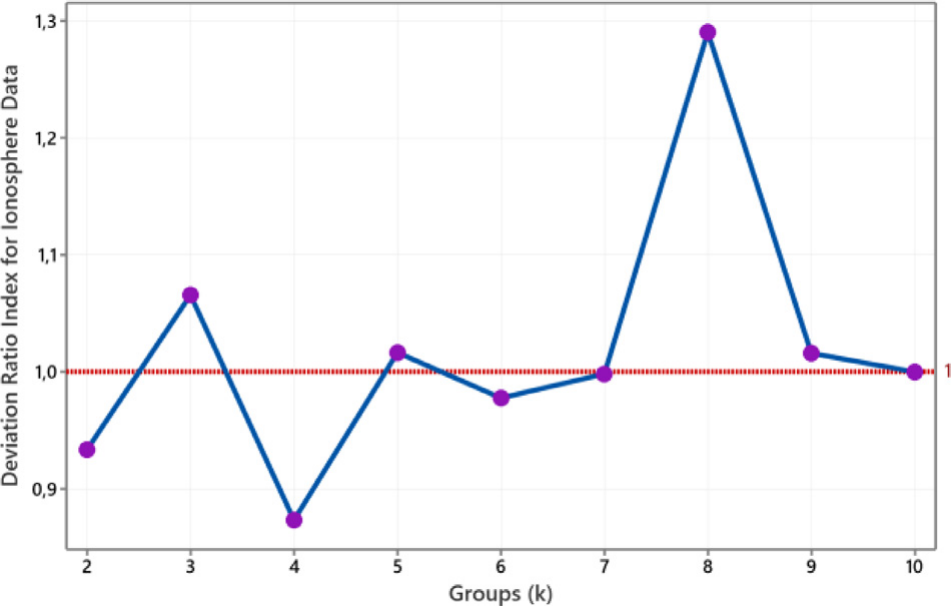
The plot of DRI for Ionosphere data.

According to Fig. 3, two groups are the smallest group sizes that produce a deviation ratio index below one. Therefore, we conclude that Breast Cancer data is classified into two groups. This size corresponds to the number of the class should be. Referring to Fig. 4, we obtain three groups for the Wine data. This size group was drawn based on the smallest group size with a DRI value of less than one achieved for three groups. The size of this group also coincides with a known group. Then Fig. 5 shows the plot of the deviation ratio index for Iris data. The smallest class that produces a DRI of less than one for Iris data is three groups conforming with real clusters. For ionosphere data, we conclude that the number of groups for this data is two, such as in Fig. 6.

**Fig. 7:**
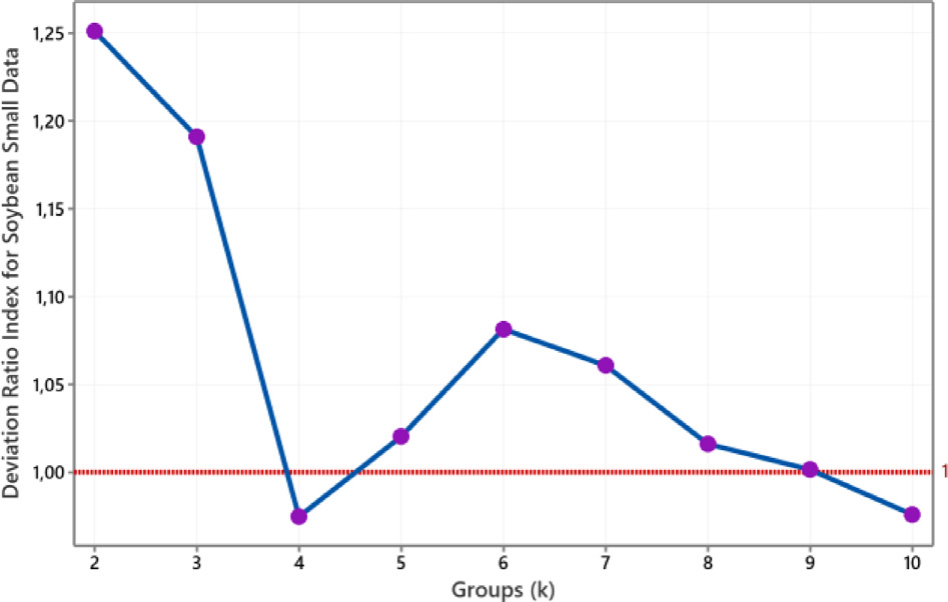
The plot of DRI for Soybean data.

**Fig. 8:**
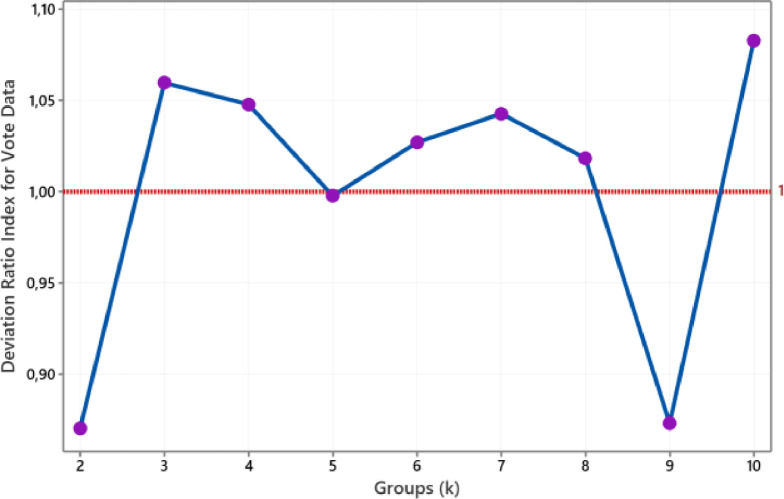
The plot of DRI for Vote data.

The value of the medoid-based deviation ratio index for Soybean (small) data is such in Fig. 7. We conclude that the optimal number of clusters for this data is four. Meanwhile, we conclude that the group size is two for categorical Vote data. This conclusion refers to the smallest group size that produces a DRI value below one is a group size of two, as shown in Fig. 8. As with fourth numerical data above, the number of clusters formed of Soybean (small) and Vote data also conform to the actual groups.

**Fig. 9:**
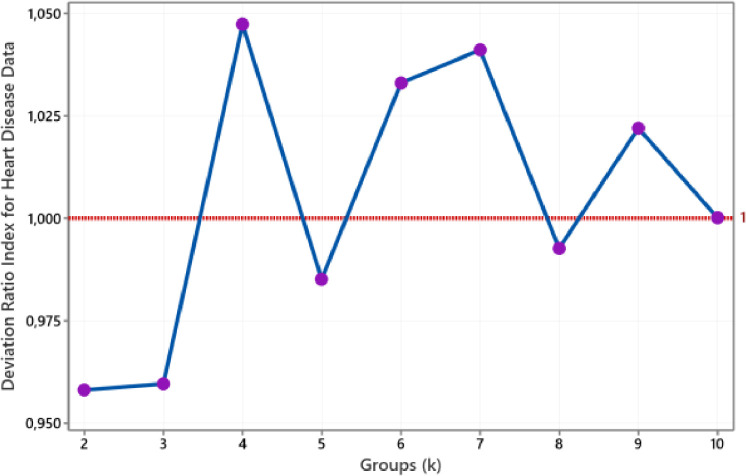
The plot of DRI for Heart Disease data.

**Fig. 10:**
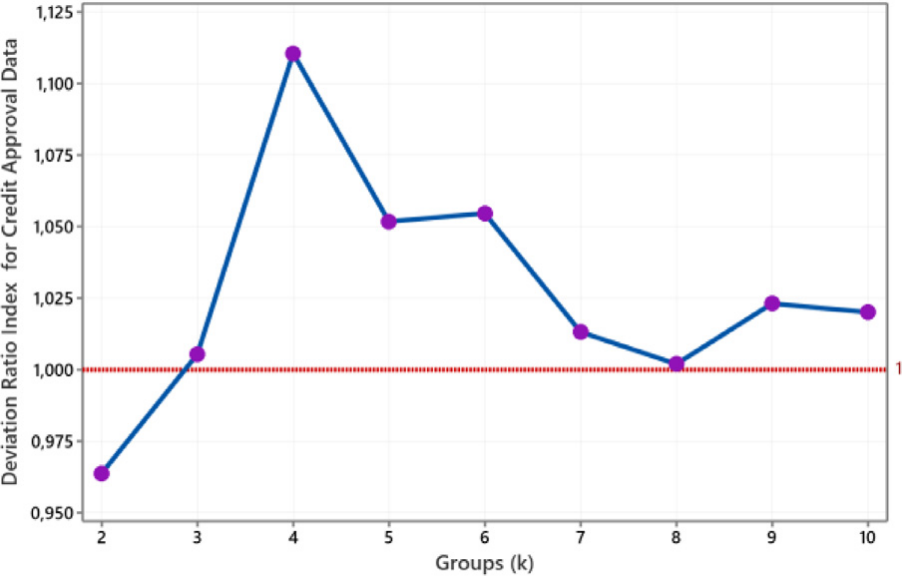
The plot of DRI for Credit Approval data.

The plot of the deviation ratio index for Heart Disease data is such in Fig. 9. We achieve the number of clusters for this data that correlates with actual clusters, i.e. two groups. Furthermore, Fig. 10 shows that the smallest group with a DRI value below one is when two groups are stamped for Credit Approval data. This size dovetails with the actual cluster.

To complete our proposed method, with the same constraint, we also calculate the maximum of the VRC and average silhouette index for eight real datasets, such as columns (6) and (7) in Table 3. We also present other methods to determine the number of clusters that uses the same actual data set, such as in column (8). The MSV focuses on visualizing and validating grouping results, not specifically on determining the number of groups. However, because this index is similar to the silhouette index [26], we try to display the group size with the highest MSV average, such as in columns (9).

**Table 3:** The number of clusters from several methods.

No	Datasets	Clustering accuracy (in per cent)	The true number of groups, k	The number of groups with several methods				k with a maximum average of MSV
				DRI (proposed)	CH index	Silhouette index	Others	
(1)	(2)	(3)	(4)	(5)	(6)	(7)	(8)	(9)
1	Breast cancer	93.5	2	**2**	2	2	4*^(a)^, 2^(d)^	2
2	Wine	91.6	3	**3**	2*	2*	4*^(a)^, 3^(b, d)^	2
3	Iris	95.3	3	**3**	2*	2*	4*^(a,c)^, 2*^(b)^,3^(d)^	2
4	Ionosphere	80.1	2	**2**	8*	2	–	2
5	Soybean	100.0	4	**4**	4	4	–	3
6	Vote	86.6	2	**2**	2	2	–	2
7	Heart disease	82.2	2	**2**	2	2	–	2
8	Credit approval	82.8	2	**2**	2	2	–	2

*missed, (a) Reference [2], (b) Reference [3], (c) Reference [8], (d) Reference [4].

According to column (3) in Table 3, we can see that the clustering accuracy of Breast Cancer, Wine, Iris, and Soybean data reaches more than 90%. The clustering accuracy produced by the BlockD-KM is classified as very compared to other grouping methods [23]. Even though the clustering accuracy of Ionosphere, Vote, Heart Disease and Credit Approval data is lower than before, we can see that the new method can precisely predict the number of groups. Meanwhile, we can see that the Calinski-Harabaz index incorrectly predicted three out of eight (37.5%) datasets. This index mispredicts the number of clusters for the Wine, Iris and Ionosphere data. Two out of eight (25%) datasets, namely Wine and Iris datasets, were also miss predicted by the silhouette index. The Krzanowski-Lai index and the PCA for determining the number of clusters also miss indicated Breast Cancer, Wine, and Iris data, such as in column (8). Suppose we use the MSV value to evaluate the number of clusters; similar to the silhouette index, the MSV value is incorrect in predicting the Wine, Iris, and Soybean data. In comparison, we can see that the new method, a medoid-based Deviation Ratio Index, can correctly predict all eight datasets.

In agreement with eight real datasets, i.e. Breast Cancer, Wine, Iris, Ionosphere, Soybean, Vote, Heart Disease, and Credit Approval data, we can see that the proposed method is better than the other methods. Though there is no satisfactory probabilistic theory to justify the use of DR(k) or DRI(k), the criterion has encouraging results, such as in column (5) in Table 3. It should be noted that using different proximities or normalization methods can produce estimates of the number of groups that may be different.

Furthermore, to evaluate medoid-based DRI, we construct three kinds of artificial datasets. The first synthetic data consists of two binary variables into two groups, which generated looks like Vote data. Then we develop the second artificial data, namely three clusters in two dimensions. The dimensions are standard normal variables with centred at (0,0), (0,5) and (5,−3). The last artificial data is a mixed variable: two binary data, one ordinal data and one numerical data. We apply the constraint as in the validation method section. The summary of the number of clusters based on the medoid-based deviation ratio index is in Table 4. Table 4 shows the number of correctly identified groups (column 8) of 115 out of 150 (76.67%) of the artificial datasets. Of the 35 incorrect decisions, 23 values were too large by 1, 8 were too large by two or more, three were too few by one, and one was too few by two or more. Accumulation of correct predictions and predictions of less than one or more than one over reached 94% (141 of 150). The number of groups determined by our proposed method is quite encouraging.

**Table 4:** The number of clusters in three artificial data.

Type	n	$p_{c}$	$p_{n}$	k	The number of trials that produce many clusters of				
					≤k−2	k−1	k	k+1	≥k+2
(1)	(2)	(3)	(4)	(5)	(6)	(7)	(8)	(9)	(10)
Categorical	100	10	–	2	n.a.	n.a.	**39**	7	4
Numerical	150	–	2	3	n.a.	0	**41**	7	2
Mixed	250	3	1	5	1	3	**35**	9	2

n.a.: not applicable.

Finally, we conclude that the newly proposed method, a medoid-based Deviation Ratio Index, is comparable to other methods. Comparisons of the DRI method with the Calinski-Harabaz index and Silhouette index on eight real datasets concluded that the new method is better than both methods. The experiment results also show that the medoid-based Deviation Ratio Index effectively determines the number of clusters. A medoid-based DRI's strength is that it is easy to calculate, suitable for all data types, applicable for small or large data sizes, and flexible for any clustering methods. It should be noted that to use the medoid-based DRI with the BlockD-KM method, the suitability in choosing the transformation method and the proximity measure will affect the prediction accuracy of the number of clusters.

## CRediT author statement

***Kariyam:*** *Conceptualization, Methodology, Coding, Validation, Data curation, Writing-Original draft preparation, Editing.*

***Abdurakhman:*** *Supervision, Validation.*

***Adhitya Ronnie Effendie*** *: Validation, Reviewing.*

## Declaration of Competing Interest

The authors declare that they have no known competing financial interests or personal relationships that could have appeared to influence the work reported in this paper.

## Data Availability

Data will be made available on request.

## Appendix Supplementary materials

Image, application 1
