# Supplementary material for: A medoid-based deviation ratio index to determine the number of clusters in a dataset
Source: MethodsX. 2023 Feb 25;10:102084. doi: 10.1016/j.mex.2023.102084 (PMC10011427; doi:10.1016/j.mex.2023.102084)
Supplement: Supplementary file 1 [file mmc1.docx]

**Supplementary material and additional information**

***Sources of data sets***

The actual dataset in Table 1 as method validation, the reader can find in Reference [12] with links below:

- Breast cancer data: <https://archive.ics.uci.edu/ml/datasets/breast+cancer+wisconsin+(diagnostic)>
- Wine data: <https://archive.ics.uci.edu/ml/datasets/wine>
- Iris data: <https://archive.ics.uci.edu/ml/datasets/iris>
- Ionosphere data: <https://archive.ics.uci.edu/ml/datasets/ionosphere>
- Soybean small data: <https://archive.ics.uci.edu/ml/datasets/soybean+(small)>
- Vote: <https://archive.ics.uci.edu/ml/datasets/congressional+voting+records>
- Heart disease: <https://archive.ics.uci.edu/ml/datasets/heart+disease>
- Credit approval: <https://archive.ics.uci.edu/ml/datasets/credit+approval>

The secondary data set in Table 2 is an illustrative example, which the reader can find in Reference [11] with links <https://epi.yale.edu/downloads/epi2022report06062022.pdf> and detail below:

- Chapter 5 Table 5-2 page 71: Regional rankings and score on Air Quality
- Chapter 6 Table 6-2 page 80: Regional rankings and score on Sanitation and Drinking Water
- Chapter 7 Table 7-2 page 87: Regional rankings and score on Heavy Metals
- Chapter 8 Table 8-2 page 95: Regional rankings and score on Waste Management
